# Supplementary material for: A python workflow definition for computational materials design
Source: Digit Discov. 2025 Oct 10;4(11):3149–61. doi: 10.1039/d5dd00231a (PMC12529651; doi:10.1039/d5dd00231a)
Supplement: DD-004-D5DD00231A-s001 [file DD-004-D5DD00231A-s001.pdf]

Supplementary Information (SI) for Digital Discovery.
This journal is © The Royal Society of Chemistry 2025

Supplementary Information for: "A Python workflow definition for computational materials design"

May 26, 2025

1 Mapping of Function Return Values

In the first version of the Python Workflow Definition (PWD), we differentiate two types of return values: Dictionaries, which enable the mapping of a specific key value pair to the input of another function, and all other types of return values, which are handled as single objects. In particular, also multi-valued types, such as tuples, lists, and arrays, are also handled as a single return object. As they are treated as single objects, the data flow between the steps of the workflow is thus fully defined via its "edges". When converting to the PWD JSON file, such non-dictionary return values are assigned to `null` ports. In the internal Python implementation, non-dictionary function return values are instead automatically wrapped in a dictionary with a default `result` key. In addition, for the use case that a Python function as part of a workflow returns a dictionary (e.g., a function imported from a third-party module, where the user doesn't have full control over the nature or construction of the returned data object) and this full dictionary is supposed to be passed in its entirety as an input to another step of the workflow, we plan to implement in a future version that another port is created with the default key `__result__` that serves to pass the entire dictionary as a single value (the mangled, dunder-name is chosen to reduce name clashes that might arise for pre-defined returned dictionaries, which might already contain a "result" entry). Thus, for the `get_prod_and_div` function from the arithmetic example of the main text, the resulting `sourcePorts` would be "prod", "div", as well as "__result__". However, we do not support this in the current version of the code, and all return dictionary key-value pairs are instead resolved to individual ports. Furthermore, based on the serialization method used in the different Workflow Management Systems (WfMS) the inputs and outputs of functions used in workflows for the PWD are restricted to data types which can be serialized using the JavaScript Object Notation (JSON). For example the atomistic structures for the calculation of the energy-versus-volume curve calculated with the Quantum ESPRESSO [5, 4] Density Functional Theory (DFT) simulation code are converted from the Atomic Simulation Environment (ASE) [6] atoms representation to the atoms JSON fromat of the OPTIMADE project [3].

2 Plot the PWD JSON graph

To assist the users in analyzing the JSON representation of the PWD, the PWD Python interface provides a `plot()` function to visualize the workflow graph:

```python
from python_workflow_definition.plot import plot
plot(file_name="arithmetic.json")
```

The workflow graph generated by the `plot()` function for the JSON representation of the arithmetic workflow above is shown in Fig. S 1. Still, in analogy to the construction of workflows, also for the visualization, it is recommended to use one of the existing WfMS as the focus of the PWD JSON format is the interoperability of the WfMS.

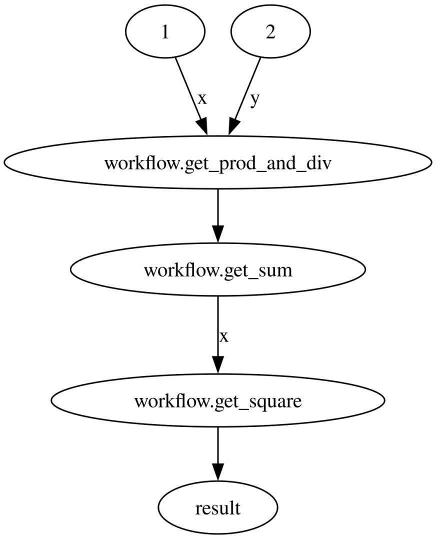

S 1: The arithmetic workflow, which computes the square of the sum of the product and quotient of two numbers 1 and 2, is visualized by the plot() function of the Python Workflow Definition Python Package based on the JSON representation of the workflow.

3 Comparison to the Abstract Syntax Tree

The mapping of the Python code representing the workflow in the PWD with its graph representation and the storage of the graph representation in the JSON format can be compared to the abstract syntax tree (AST) [1] of the Python standard library. The AST is the internal representation of the parsed Python code during execution with the Python interpreter. When executing a Python program from a file, the Python interpreter reads the input file line by line and constructs a hierarchical graph, called AST, of which lines should be executed in which order. In analogy, the PWD constructs a graph for executing different workflow steps represented as nodes and connected by edges to other nodes. Still, the important distinction between the Python AST and the PWD is the focus on interoperability between different WfMS. With the PWD, the same workflow defined in either AiiDA, jobflow, or pyiron is represented by the same workflow graph, resulting in the same JSON representation. In contrast their corresponding AST graph is WfMS specific, as the Python commands of the WfMS are also parsed by the AST. Consequently, the advantage of the PWD is that a workflow defined in one WfMS can be exported to any other WfMS. In contrast, this is not possible with the Python AST, as the WfMS themselves use Python internally, resulting in different representations in the Python AST.

4 Energy-Versus-Volume Curve Workflow

The energy-versus-volume curve workflow is commonly used in combination with Density Functional Thoery (DFT) simulation codes to compute the equilibrium bulk modulus and equilibrium volume. The same workflow is implemented with AiiDA, jobflow and pyiron to highlight the similarities of these WfMS, which all use the Python programming language as workflow language. Following the construction of the workflow in one WfMS the workflow graph is stored in the JSON to be reloaded with any of the other two WfMS as demonstrated in the manuscript. The python modules `python_workflow_definition` and `quantum_espresso_workflow` are available in the Github repository of the PWD.

4.1 AiiDA

```
from aiida import load_profile, orm
load_profile()
```

```python
from aiida_workgraph import task, WorkGraph
from python_workflow_definition.aiida import write_workflow_json
from python_workflow_definition.shared import get_dict, get_list
from quantum_espresso_workflow import (
    generate_structures,
    get_bulk_structure,
    calculate_qe as _calculate_qe,
    plot_energy_volume_curve,
)

calculate_qe = task(outputs=["energy", "volume", "structure"])(_calculate_qe)

wg = WorkGraph("wg-qe")

element = orm.Str("Al")
a = orm.Float(4.04)
cubic = orm.Bool(True)
relax_workdir = orm.Str("mini")
pseudopotentials = orm.Dict({"Al": "Al.pbe-n-kjpaw_psl.1.0.0.UPF"})
kpts = orm.List([3, 3, 3])
calc_type_relax = orm.Str("vc-relax")
calc_type_scf = orm.Str("scf")
smearing = orm.Float(0.02)
strain_lst = orm.List([0.9, 0.95, 1.0, 1.05, 1.1])

get_bulk_structure_task = wg.add_task(
    get_bulk_structure,
    element=element,
    a=a,
    cubic=cubic,
)

relax_prepare_input_dict_task = wg.add_task(
    get_dict,
    structure=get_bulk_structure_task.outputs.result,
    calculation=calc_type_relax,
    kpts=kpts,
    pseudopotentials=pseudopotentials,
    smearing=smearing,
)

relax_task = wg.add_task(
    calculate_qe,
    input_dict=relax_prepare_input_dict_task.outputs.result,
    working_directory=relax_workdir,
)

generate_structures_task = wg.add_task(
    generate_structures,
    structure=relax_task.outputs.structure,
    strain_lst=strain_lst,
)

```

```
get_volumes_task = wg.add_task(get_list)
get_energies_task = wg.add_task(get_list)

strain_dir_tasks, scf_qe_tasks, scf_get_dict_tasks = [], [], []

for i, strain in enumerate(strain_lst):

    structure_key = f"s_{i}"
    strain_dir = orm.Str(f"strain_{i}")
    generate_structures_task.add_output("workgraph.any", structure_key)

    scf_prepare_input_dict_task = wg.add_task(
        get_dict,
        structure=generate_structures_task.outputs[structure_key],
        calculation=calc_type_scf,
        kpts=kpts,
        pseudopotentials=pseudopotentials,
        smearing=smearing,
    )

    scf_qe_task = wg.add_task(
        calculate_qe,
        input_dict=scf_prepare_input_dict_task.outputs.result,
        working_directory=strain_dir,
    )

    get_energies_task.set({f"{i}": scf_qe_task.outputs.energy})
    get_volumes_task.set({f"{i}": scf_qe_task.outputs.volume})

plot_energy_volume_curve_task = wg.add_task(
    plot_energy_volume_curve,
    volume_lst=get_volumes_task.outputs.result,
    energy_lst=get_energies_task.outputs.result,
)

write_workflow_json(wg=wg, file_name="aiida_qe.json")
```

4.2 jobflow

```
import numpy as np
from jobflow import job, Flow
from python_workflow_definition.jobflow import write_workflow_json
from quantum_espresso_workflow import (
    calculate_qe as _calculate_qe,
    generate_structures as _generate_structures,
    get_bulk_structure as _get_bulk_structure,
    plot_energy_volume_curve as _plot_energy_volume_curve,
)

calculate_qe = job(_calculate_qe, data=["energy", "volume", "structure"])
generate_structures = job(_generate_structures, data=[f"s_{i}" for i in range(100)])
plot_energy_volume_curve = job(_plot_energy_volume_curve)
get_bulk_structure = job(_get_bulk_structure)

```

```python
pseudopotentials = {"Al": "Al.pbe-n-kjpaw_psl.1.0.0.UPF"}

structure = get_bulk_structure(
    element="Al",
    a=4.04,
    cubic=True,
)

calc_mini = calculate_qe(
    working_directory="mini",
    input_dict={
        "structure": structure.output,
        "pseudopotentials": pseudopotentials,
        "kpts": (3, 3, 3),
        "calculation": "vc-relax",
        "smearing": 0.02,
    },
)

number_of_strains = 5
structure_lst = generate_structures(
    structure=calc_mini.output.structure,
    strain_lst=np.linspace(0.9, 1.1, number_of_strains),
)

job_strain_lst = []
for i in range(number_of_strains):
    calc_strain = calculate_qe(
        working_directory="strain_" + str(i),
        input_dict={
            "structure": getattr(structure_lst.output, f"s_{i}"),
            "pseudopotentials": pseudopotentials,
            "kpts": (3, 3, 3),
            "calculation": "scf",
            "smearing": 0.02,
        },
    )
    job_strain_lst.append(calc_strain)

plot = plot_energy_volume_curve(
    volume_lst=[job.output.volume for job in job_strain_lst],
    energy_lst=[job.output.energy for job in job_strain_lst],
)

flow = Flow([structure, calc_mini, structure_lst] + job_strain_lst + [plot])
write_workflow_json(flow=flow, file_name="jobflow_qe.json")
```

4.3 pyiron

```python
import numpy as np
from pyiron_base import job
from python_workflow_definition.pyiron_base import write_workflow_json
from quantum_espresso_workflow import (
    calculate_qe as _calculate_qe,
```

```
    generate_structures as _generate_structures,
    get_bulk_structure as _get_bulk_structure,
    plot_energy_volume_curve as _plot_energy_volume_curve,
)

calculate_qe = job(_calculate_qe, output_key_lst=["energy", "volume", "structure"])
generate_structures = job(_generate_structures)
plot_energy_volume_curve = job(_plot_energy_volume_curve)
get_bulk_structure = job(_get_bulk_structure)

pseudopotentials = {"Al": "Al.pbe-n-kjpaw_psl.1.0.0.UPF"}

structure = get_bulk_structure(
    element="Al",
    a=4.04,
    cubic=True,
)

calc_mini = calculate_qe(
    working_directory="mini",
    input_dict={
        "structure": structure,
        "pseudopotentials": pseudopotentials,
        "kpts": (3, 3, 3),
        "calculation": "vc-relax",
        "smearing": 0.02,
    },
)

number_of_strains = 5
structure_lst = generate_structures(
    structure=calc_mini.output.structure,
    strain_lst=np.linspace(0.9, 1.1, number_of_strains),
    list_length=number_of_strains,
)

job_strain_lst = []
for i, structure_strain in enumerate(structure_lst):
    calc_strain = calculate_qe(
        working_directory="strain_" + str(i),
        input_dict={
            "structure": structure_strain,
            "pseudopotentials": pseudopotentials,
            "kpts": (3, 3, 3),
            "calculation": "scf",
            "smearing": 0.02,
        },
    )
    job_strain_lst.append(calc_strain)

plot = plot_energy_volume_curve(
    volume_lst=[job.output.volume for job in job_strain_lst],
    energy_lst=[job.output.energy for job in job_strain_lst],
)
```

```python

write_workflow_json(delayed_object=plot, file_name="pyiron_qe.json")
```

5 File-based Workflow

The finite element file-based workflow was initially published in [2]. It is used here primarily as a demonstration to highlight the application of the PWD to file-based workflows. The PWD python module and `nfdi_ing_workflow`, which are reused by all WfMS, are available in the PWD Github repository.

5.1 AiiDA

```python
from aiida import orm, load_profile
load_profile()

from aiida_workgraph import WorkGraph, task
from python_workflow_definition.aiida import write_workflow_json
from nfdi_ing_workflow import (
    generate_mesh,
    convert_to_xdmf as _convert_to_xdmf,
    poisson as _poisson,
    plot_over_line,
    substitute_macros,
    compile_paper,
)

convert_to_xdmf = task(outputs=["xdmf_file", "h5_file"])(_convert_to_xdmf)
poisson = task(outputs=["numdofs", "pvd_file", "vtu_file"])(_poisson)

domain_size = orm.Float(2.0)

wg = WorkGraph("wg-nfdi")

gmsh_output_file = wg.add_task(
    generate_mesh,
    domain_size=domain_size,
)

meshio_output_dict = wg.add_task(
    convert_to_xdmf,
    gmsh_output_file=gmsh_output_file.outputs.result,
)

poisson_dict = wg.add_task(
    poisson,
    meshio_output_xdmf=meshio_output_dict.outputs.xdmf_file,
    meshio_output_h5=meshio_output_dict.outputs.h5_file,
)

pvbatch_output_file = wg.add_task(
    plot_over_line,
    poisson_output_pvd_file=poisson_dict.outputs.pvd_file,
    poisson_output_vtu_file=poisson_dict.outputs.vtu_file,
)

```

```python
macros_tex_file = wg.add_task(
    substitute_macros,
    pvbatch_output_file=pvbatch_output_file.outputs.result,
    ndofs=poisson_dict.outputs.numdofs,
    domain_size=domain_size,
)

paper_output = wg.add_task(
    compile_paper,
    macros_tex=macros_tex_file.outputs.result,
    plot_file=pvbatch_output_file.outputs.result,
)

write_workflow_json(wg=wg, file_name="aiida_nfdi.json")
```

5.2 jobflow

```python
from jobflow import job, Flow
from python_workflow_definition.jobflow import write_workflow_json
from nfdi_ing_workflow import (
    generate_mesh as _generate_mesh,
    convert_to_xdmf as _convert_to_xdmf,
    poisson as _poisson,
    plot_over_line as _plot_over_line,
    substitute_macros as _substitute_macros,
    compile_paper as _compile_paper,
)

generate_mesh = job(_generate_mesh)
convert_to_xdmf = job(_convert_to_xdmf, data=["xdmf_file", "h5_file"])
poisson = job(_poisson, data=["numdofs", "pvd_file", "vtu_file"])
plot_over_line = job(_plot_over_line)
substitute_macros = job(_substitute_macros)
compile_paper = job(_compile_paper)

domain_size = 2.0

gmsh_output_file = generate_mesh(
    domain_size=domain_size,
)

meshio_output_dict = convert_to_xdmf(
    gmsh_output_file=gmsh_output_file.output,
)

poisson_dict = poisson(
    meshio_output_xdmf=meshio_output_dict.output.xdmf_file,
    meshio_output_h5=meshio_output_dict.output.h5_file,
)

pvbatch_output_file = plot_over_line(
    poisson_output_pvd_file=poisson_dict.output.pvd_file,
    poisson_output_vtu_file=poisson_dict.output.vtu_file,
)
```

```
macros_tex_file = substitute_macros(
    pvbatch_output_file=pvbatch_output_file.output,
    ndofs=poisson_dict.output.numdofs,
    domain_size=domain_size,
)

paper_output = compile_paper(
    macros_tex=macros_tex_file.output,
    plot_file=pvbatch_output_file.output,
)

flow = Flow([
    gmsh_output_file,
    meshio_output_dict,
    poisson_dict,
    pvbatch_output_file,
    macros_tex_file,
    paper_output,
])
write_workflow_json(flow=flow, file_name="jobflow_nfdi.json")
```

5.3 pyiron

```
from nfdi_ing_workflow import (
    generate_mesh as _generate_mesh,
    convert_to_xdmf as _convert_to_xdmf,
    poisson as _poisson,
    plot_over_line as _plot_over_line,
    substitute_macros as _substitute_macros,
    compile_paper as _compile_paper,
)
from pyiron_base import job
from python_workflow_definition.pyiron_base import write_workflow_json

generate_mesh = job(_generate_mesh)
convert_to_xdmf = job(_convert_to_xdmf, output_key_lst=["xdmf_file", "h5_file"])
poisson = job(_poisson, output_key_lst=["numdofs", "pvd_file", "vtu_file"])
plot_over_line = job(_plot_over_line)
substitute_macros = job(_substitute_macros)
compile_paper = job(_compile_paper)

domain_size = 2.0

gmsh_output_file = generate_mesh(
    domain_size=domain_size,
)

meshio_output_dict = convert_to_xdmf(
    gmsh_output_file=gmsh_output_file,
)

poisson_dict = poisson(
    meshio_output_xdmf=meshio_output_dict.output.xdmf_file,
```

```
    meshio_output_h5=meshio_output_dict.output.h5_file,
)

pvbatch_output_file = plot_over_line(
    poisson_output_pvd_file=poisson_dict.output.pvd_file,
    poisson_output_vtu_file=poisson_dict.output.vtu_file,
)

macros_tex_file = substitute_macros(
    pvbatch_output_file=pvbatch_output_file,
    ndofs=poisson_dict.output.numdofs,
    domain_size=domain_size,
)

paper_output = compile_paper(
    macros_tex=macros_tex_file,
    plot_file=pvbatch_output_file,
)

write_workflow_json(delayed_object=paper_output, file_name="pyiron_nfdi.json")
```

References

[1] Abstract syntax trees. `https://docs.python.org/3/library/ast.html`. Accessed: 2025-05-21.

[2] Philipp Diercks, Dennis Gläser, Ontje Lünsdorf, Michael Selzer, Bernd Flemisch, and Jörg F. Unger. Evaluation of tools for describing, reproducing and reusing scientific workflows. *ing.grid*, 1, 8 2023.

[3] Matthew L. Evans, Johan Bergsma, Andrius Merkys, Casper W. Andersen, Oskar B. Andersson, Daniel Beltrán, Evgeny Blokhin, Tara M. Boland, Rubén Castañeda Balderas, Kamal Choudhary, Alberto Díaz Díaz, Rodrigo Domínguez García, Hagen Eckert, Kristjan Eimre, María Elena Fuentes Montero, Adam M. Krajewski, Jens Jørgen Mortensen, José Manuel Nápoles Duarte, Jacob Pietryga, Ji Qi, Felipe de Jesús Trejo Carrillo, Antanas Vaitkus, Jusong Yu, Adam Zettel, Pedro Baptista de Castro, Johan Carlsson, Tiago F. T. Cerqueira, Simon Divilov, Hamidreza Hajiyani, Felix Hanke, Kevin Jose, Corey Oses, Janosh Riebesell, Jonathan Schmidt, Donald Winston, Christen Xie, Xiaoyu Yang, Sara Bonella, Silvana Botti, Stefano Curtarolo, Claudia Draxl, Luis Edmundo Fuentes Cobas, Adam Hospital, Zi-Kui Liu, Miguel A. L. Marques, Nicola Marzari, Andrew J. Morris, Shyue Ping Ong, Modesto Orozco, Kristin A. Persson, Kristian S. Thygesen, Chris Wolverton, Markus Scheidgen, Cormac Toher, Gareth J. Conduit, Giovanni Pizzi, Saulius Gražulis, Gian-Marco Rignanese, and Rickard Armiento. Developments and applications of the OPTIMADE API for materials discovery, design, and data exchange. *Digital Discovery*, 3:1509–1533, 2024.

[4] P Giannozzi, O Andreussi, T Brumme, O Bunau, M Buongiorno Nardelli, M Calandra, R Car, C Cavazzoni, D Ceresoli, M Cococcioni, N Colonna, I Carnimeo, A Dal Corso, S de Gironcoli, P Delugas, R A DiStasio, A Ferretti, A Floris, G Fratesi, G Fugallo, R Gebauer, U Gerstmann, F Giustino, T Gorni, J Jia, M Kawamura, H-Y Ko, A Kokalj, E Küçükbenli, M Lazzeri, M Marsili, N Marzari, F Mauri, N L Nguyen, H-V Nguyen, A Otero-de-la Roza, L Paulatto, S Poncé, D Rocca, R Sabatini, B Santra, M Schlipf, A P Seitsonen, A Smogunov, I Timrov, T Thonhauser, P Umari, N Vast, X Wu, and S Baroni. Advanced capabilities for materials modelling with quantum espresso. *Journal of Physics: Condensed Matter*, 29(46):465901, 10 2017.

[5] Paolo Giannozzi, Stefano Baroni, Nicola Bonini, Matteo Calandra, Roberto Car, Carlo Cavazzoni, Davide Ceresoli, Guido L Chiarotti, Matteo Cococcioni, Ismaila Dabo, Andrea Dal Corso, Stefano de Gironcoli, Stefano Fabris, Guido Fratesi, Ralph Gebauer, Uwe Gerstmann, Christos Gougoussis, Anton Kokalj, Michele Lazzeri, Layla Martin-Samos, Nicola Marzari, Francesco Mauri, Riccardo Mazzarello, Stefano

Paolini, Alfredo Pasquarello, Lorenzo Paulatto, Carlo Sbraccia, Sandro Scandolo, Gabriele Sclauzero, Ari P Seitsonen, Alexander Smogunov, Paolo Umari, and Renata M Wentzcovitch. Quantum espresso: a modular and open-source software project for quantum simulations of materials. *Journal of Physics: Condensed Matter*, 21(39):395502, 9 2009.

[6] Ask Hjorth Larsen, Jens Jørgen Mortensen, Jakob Blomqvist, Ivano E Castelli, Rune Christensen, Marcin Dułak, Jesper Friis, Michael N Groves, Bjørk Hammer, Cory Hargus, Eric D Hermes, Paul C Jennings, Peter Bjerre Jensen, James Kermode, John R Kitchin, Esben Leonhard Kolsbjerg, Joseph Kubal, Kristen Kaasbjerg, Steen Lysgaard, Jón Bergmann Maronsson, Tristan Maxson, Thomas Olsen, Lars Pastewka, Andrew Peterson, Carsten Rostgaard, Jakob Schiøtz, Ole Schütt, Mikkel Strange, Kristian S Thygesen, Tejs Vegge, Lasse Vilhelmsen, Michael Walter, Zhenhua Zeng, and Karsten W Jacobsen. The atomic simulation environment—a python library for working with atoms. *Journal of Physics: Condensed Matter*, 29(27):273002, 06 2017.
